# Micronutrient Intake in Healthy Toddlers: A Multinational Perspective

**DOI:** 10.3390/nu7085316

**Published:** 2015-08-18

**Authors:** Jennifer Hilger, Tatiana Goerig, Peter Weber, Birgit Hoeft, Manfred Eggersdorfer, Nina Costa Carvalho, Ursula Goldberger, Kristina Hoffmann

**Affiliations:** 1 Mannheim Institute of Public Health, Social and Preventive Medicine, Medical Faculty Mannheim, Heidelberg University, Ludolf-Krehl-Strasse 7-11, Mannheim 68167, Germany; E-Mails: Jennifer.Hilger@medma.uni-heidelberg.de (J.H.); Tatiana.Goerig@medma.uni-heidelberg.de (T.G.); Nina.Costa-Carvalho@medma.uni-heidelberg.de (N.C.C.); Ursula.Goldberger@medma.uni-heidelberg.de (U.G.); 2 DSM Nutritional Products Limited, Wurmisweg 576, Kaiseraugst 4303, Switzerland; E-Mails: Peter.Weber@dsm.com (P.W.); Birgit.Hoeft@dsm.com (B.H.); Manfred.Eggersdorfer@dsm.com (M.E.)

**Keywords:** child, preschool, nutrition assessment, nutrient intake, nutritional status, micronutrients

## Abstract

Adequate nutrient intake during early childhood is of particular importance for optimal growth and future health. However, cross-national comparative research on nutrient intake of toddlers is still limited. We conducted a literature review to examine the nutrient intake in healthy toddlers from some of the world’s most populous nations currently on different stages of socioeconomic development: Brazil, Germany, Russia and the United States. We aimed to identify national surveys reporting mean intakes of the following nutrients: vitamins A, D, E, folate, calcium, iron and zinc. To calculate the prevalence of inadequate nutrient intake, we used a modified version of the Estimated Average Requirement cut-point method. Overall, five studies with 6756 toddlers were eligible for inclusion in this review. In countries where data were available, a prevalence of inadequate intake higher than 20% was found for vitamins A, D, E and calcium. In Germany, folate intake also appeared to be inadequate. The results of our review indicate that inadequate micronutrient intake in toddlers might be a global challenge affecting also affluent countries. However, to explore the full scope of this important public health issue joint efforts of researchers worldwide are needed to combine existing data and fill in data gaps.

## 1. Introduction

The toddler years, from one to three years of life, are characterized by crucial physical, mental and behavioral changes, such as rapid growth, weight gain, the development of cognitive and psychomotor skills, and a transition in food preferences and eating habits [1]. On a per kilogram of body weight basis, toddlers’ daily nutrient requirements are higher than those of older children and adults, but their gastric capacity is only about one-third that of adults [2]. Therefore toddlers need small but energy- and nutrient-dense meals throughout the day to meet their basal requirements [2,3].

Culture and traditional cuisines, along with food supply and availability of specific types of food, reflect the socioeconomic development of a country and influence what parents feed their toddlers [4,5]. In the developing world, where a diverse supply of foods is limited, micronutrient deficiencies in young children are a major public health problem [6]. However, recent studies indicate that inadequate intake of one or more micronutrients is also a public health issue in transitional economies, such as the former Soviet Union states, and in affluent countries like Germany and the United States [7,8,9,10]. Although evidence is increasing that early childhood nutrition might affect adult morbidity and mortality, there is still a lack of comparative research with regard to the nutrient intake of toddlers.

We therefore conducted a literature review on the nutrient intake in healthy toddlers from Brazil, Germany, Russia and the United States. According to the Global Competiveness Index these four countries are currently in different stages of socioeconomic development [11]. In addition, these countries represent the most populous nations of the following world regions: Latin America, Western Europe, Eastern Europe and North America, respectively. Nutrient intake patterns identified in healthy toddlers across these different nations might also be present in other nations from the same world region or in countries at similar stages of socioeconomic development. Thus, identification of such patterns might be helpful to support pediatricians, nutritionists, public health researchers and policy makers in implementing strategies to prevent nutrient deficiencies during the first years of life. The specific aims of our review were: (a) to provide an overview of available studies on the intake of selected nutrients in children aged one to three years; (b) to compare nutrient intake in this age group across these countries; and (c) to assess cross-national patterns of inadequate nutrient intake.

## 2. Material and Methods

### 2.1. Eligibility Criteria

Studies were eligible if they met the inclusion criteria defined *a priori* (Table 1). Given their importance to the development of multiple organ systems throughout childhood and their possible benefits on future health, the outcome of interest was the mean intake of vitamins A, D, E, folate, calcium, iron, and zinc.

**Table 1:** Inclusion criteria for studies eligible for the review.

**Characteristics**	
**populations**	Samples of healthy toddlers (1–3 years)
	randomly selected from the general population;
	recruited in clinical setting (e.g., during a pediatric health check-up);
	recruited in day-care facilities (e.g., preschools);
	recruited via their mothers (e.g., at delivery);
	and (if reported) population subgroups by age and sex
**outcomes**	Mean intake value of at least one of the following nutrients:
	- vitamin A
	- vitamin D
	- vitamin E
	- folate
	- calcium
	- iron
	- zinc
**countries**	- Brazil
	- Germany
	- Russia
	- United States
**study designs**	- Cross-sectional studies
	- (Population-based) cohort studies (latest data)
**publication date**	- Between 2000/01/01 and 2014/01/15

### 2.2. Search Strategy

To identify eligible studies on the nutritional intake status of healthy toddlers for each country, we used the Pubmed/Medline database as the primary source for original survey-based research articles. We chose the following keywords from the Medical Subject Heading (MeSH) thesaurus: “child, preschool”, “nutrition assessment”, “nutritional status”. These keywords as well as the free text term “toddler” and the country name were searched. The search was restricted to articles published from 1 January 2000 to 15 January 2014 (date of final screen). Articles published before this timeframe were excluded due to important changes in diet. These changes may have occurred with industrialization, economic development and market globalization, thereby introducing bias in the assessment of the nutrient intake of those living in economies in transition [12].

Additionally, the following databases were searched using keywords in English and in the national language of the country: (a) Literatura Latino Americana e do Caribe em Ciências da Saúde (LILACS) referencing publications in Portuguese, (b) *Elibrary* (http://www.elibrary.ru/) and the electronic catalogue *Medicine*, accessed via Central Scientific Medical Library referencing publications in Russian, (c) “Google” and (d) “Google Scholar”. Reference lists of identified articles were screened to detect other articles which might be eligible.

To identify survey data from non-peer-reviewed studies or reports, sometimes referred to as “grey” literature, we also used “Google” and “Google Scholar”. In addition, we searched the websites of national nutrition societies (e.g., German Nutrition Society), research institutions (e.g., the World Health Organization (WHO), the Academy of Nutrition and Dietetics) and governmental organizations (e.g., Ministry of Health Protection of the Russian Federation). If studies reported on nutrients of interest but did not provide data in the required format (e.g., data were reported in percentages and not as mean values), we contacted the corresponding authors and asked for the original data. The detailed search strategy and the exact search query for each country are provided in supplement.

Four of the authors (J.H., T.G., N.C.C., and U.G.) independently searched, selected and reviewed potentially eligible studies for this review. Decisions on study eligibility for review were made by consensus.

### 2.3. Data Extraction and Data Elements

Data were extracted using a standardized data extraction form, which included intake values of the selected nutrients, intake assessment method (e.g., dietary record, 24-hour recall) and dietary analysis programs (e.g., Nutrition Data System for Research 2008). In addition, we documented characteristics of the study population, the method of recruitment (e.g., recruitment at preschools or by use of household panels) and statements made by the authors on the representativeness of the samples or the potential for selection bias. In some instances, data on the same populations were reported in multiple articles. In these cases, only those providing complementary information (e.g., one reported value for vitamin A and the other for vitamin D) were considered for analysis.

### 2.4. Determination of an Adequate Nutrient Intake and Data Analysis

For estimating the prevalence of inadequate nutrient intakes within a group, the Institute of Medicine (IOM) recommends amongst others the Estimated Average Requirement (EAR) cut-point method [13]. A modified version of this method developed by the European Micronutrient Recommendations Aligned Network of Excellence (EURRECA) was employed, allowing the use of aggregated data [14]. To apply the EAR cut-point method, the requirement distribution of the nutrient intake must follow a symmetrical distribution [13].

During the first step of the method, we calculated the EAR cut-point. The sample mean nutrient intakes were *z*-transformed according to the following formula: *z* = (*x* − *µ*)/SD, where *x* is the EAR reference value established by the IOM, *µ* the sample mean nutrient intake and SD the corresponding standard deviation [14]. During the next step we estimated the proportion within the population which fell below this cut-point by using the *normprob* command in Stata (Stata Corp., TX, US, Version 12.1) and multiplying the result by 100. This proportion represents the prevalence of inadequate nutrient intakes within the group (% below EAR).

We had to define a threshold to evaluate the adequacy of nutrient intakes for the studies included. Although the WHO recommends 2%–3% as the maximum desirable prevalence of inadequate intake [15], we decided to use a more permissive threshold value because we did not have access to the original data. Consequently, we set a value below 20% as the maximum desirable prevalence of inadequate intake, which is in line with a previous study also using aggregated data [14].

Descriptive statistics for baseline characteristics of all included studies were calculated using Stata 12.1 (Stata Corp., College Station, TX, USA).

## 3. Results

### 3.1. Overview of Available Studies

Overall, five studies with a total of 6756 children (sample size range: 488 to 2386 participants) were eligible for inclusion in this review (Table 2). Data reported in seven publications [16,17,18,19,20,21,22] were considered. We identified at least one study which met our predefined inclusion criteria for each country of interest; two of these countries reported recruitment of representative samples. We noted that the method of dietary assessment (e.g., 24-hour recall, dietary record) differed across studies and that in some articles the focus was on specific subgroups defined by age or sex. Supplement intake was specifically assessed in a single study from the United States [17]. For vitamin D mean intake data were available for Brazil and the United States but not for Germany and Russia. Russian studies examining intake of vitamins E, folate or zinc could also not be identified for this age group. No cross-national comparisons for adequate intakes of vitamins A, D, or E with Brazilian toddlers could be made using the EAR cut-point method due to reports that the distribution of these nutrients in the Nutri-Brasil Infância study was skewed [18].

### 3.2. Comparison of Nutrient Intakes in Healthy Toddlers from Brazil, Germany, Russia and the United States

Cross-national comparisons suggested similar mean intake values for vitamin A, folate, iron and zinc for toddlers from the United States and Brazil (Table 3, Table 4, Table 5, Table 6, Table 7, Table 8 and Table 9). The highest mean intake values of vitamin D, calcium and vitamin E (only in the Feeding Infants and Toddlers Study (FITS)) were reported in US toddlers. In most instances, the mean nutrient intake among German toddlers was lower compared to those in the United States and Brazil, particularly for folate. Although reports of mean vitamin A intake were similar in German and Russian studies, mean intake of calcium and iron in Germany was lower.

**Table 2:** Characteristics of the studies included in the review.

Country	Time of Data Collection	Population *n*	Selection of Participants	Dietary Assessment Method	Dietary Assessment Program/Nutrient Database	Intake Includes Supplements	Selection Bias (as Reported by the Authors)	Nutrients Not Reported
**Brazil**								
Nutri-Brasil Infância [18]	February–December 2007	1689	Multicenter study (9 cities); subjects were recruited at public and private daycare centers, preschools	Two Dietary records: one-day weighed food records were used to assess the intake at school mealtime; one-day estimated food records were completed by the parents to assess school dietary intake	Nutrition Data System for Research 2007 (NDSR 2007); Brazilian Food Composition Table (for regional foods)	Unknown	The study used a non-representative sample	None
**Germany**								
VELS Study [19,20]	June 2001–September 2002	488	Multicenter study (10 cities); subjects were recruited at places frequently attended by parents of toddlers (e.g., mothers and toddlers groups, preschools, day-care centers)	Dietary record: two three-day dietary records were completed by the parents	EAT 2002 (based on the BLS II.3 and the LEBTAB database from the German DONALD Study)	No	Children of parents with higher educational background were overrepresented	Vitamin D
**Russia**								
Healthy development from first days of life [16]	2006 (not further defined)	1404	The study was performed in 38 Russian regions using the Russian Representative Sampling	24-hour recall	Computer database containing information on 3000 products and dishes	Unknown	Unknown	Vitamins D, E, folate, zinc
**United States**									
FITS [17]	June 2008–January 2009	2386	Participants were randomly selected from two databases of commercial vendor; Databases were supposed to form a national sample frame.	24-hour recall: the primary caregiver of each child Participated in one 24-hour recall	Nutrition Data System for Research 2008 (NDSR 2008)	Yes	Hispanics and non-Hispanic blacks were underrepresented	None
NHANES [21,22]	January 2001–December 2002; January 2005–December 2006	798	A complex, stratified, multistage probability cluster sampling design was used.	24-hour recall: the primary caregiver participated in two 24-hour recalls (first one conducted in-person; second one conducted over the telephone)	USDA Food and Nutrient Database for Dietary Studies 3.0 (FNDDS 3.0)	No	National representative survey of the civilian non-institutionalized U.S. population	None

VELS, food composition survey to determine food intake by infants and small children for the estimation of acute toxicity risk from pesticide residues; FITS, feeding infants and toddlers study; NHANES, National Health and Nutrition Examination Survey.

### 3.3. Cross-National Comparison of Nutrient Intake Adequacy Estimated by the EAR Cut-point Method

Using the modified EAR cut-point method to assess the prevalence of inadequate nutrient intake, inadequacies across all four countries could be identified (Table 3, Table 4, Table 5, Table 6, Table 7, Table 8 and Table 9). A prevalence of inadequate intake higher than 20% was seen for vitamin A in Germany and Russia as well as for two-year-olds in the National Health and Nutrition Examination Survey (NHANES) from the United States (Table 3). Additionally, a prevalence of inadequate intake higher than 20% could be found for vitamin E in Germany and for vitamins E and D in the United States (Table 4 and Table 5).

In Germany, inadequate intakes could also be found for folate across all age and sex groups (Table 6). For example, about 49.0% of the two-year-old boys and girls here had folate intakes below the EAR. In the remaining studies prevalence of an inadequate folate intake varied between 3.1%, and 19.9%. Calcium intakes in Germany (between 31.8% and 39.0% below EAR in different subgroups) and Russia (29.7% below EAR) as well as the USA (only NHANES: 31.9%) were found to be inadequate (Table 7). In the Brazilian Nutri-Brasil Infância study this was only the case for toddlers which were two years and older. The intake values for iron were found to be adequate in all four countries (Table 8). This was also seen for zinc in all countries besides Russia, where no data on zinc intake were available for toddlers (Table 9).

**Table 3:** Mean vitamin A intake (mg/day) and prevalence of inadequate intake (% below EAR) in Brazil, Germany, Russia and the United States.

Country	Study Name	Age Range (year)	Sex	*n*	EAR (mg/day)	Mean (mg/day)	SD (mg/day)	% below EAR (95% CI)
Brasil	Nutri Brazil Infantia [18]	1.0	m + f	58	**0.21**	0.67	0.58	no assessment possible
		2.0	m + f	657	**0.21**	0.97	1.83	no assessment possible
		3.0	m + f	974	**0.21**	0.79	1.20	no assessment possible
Germany	VELS [19,20]	1.0–1.9	m	81	**0.21**	0.59	0.49	21.90 (20.89; 22.92)
		1.0–1.9	f	87	**0.21**	0.62	0.61	25.08 (24.08; 26.07)
		2.0–2.9	m	89	**0.21**	0.71	0.7	23.75 (22.80; 24.70)
		2.0–2.9	f	85	**0.21**	0.58	0.61	27.21 (26.17; 28.25)
		3.0–3.9	m	71	**0.21**	0.75	0.83	25.77 (24.54; 26.99)
		3.0–3.9	f	74	**0.21**	0.61	0.53	22.52 (21.40; 23.65)
Russia	Healthy development from the first days of life [16]	1.0–2.0	m + f	1404	**0.21**	0.65	1.01	33.16 (33.10; 33.23)
USA	FITS [17]	1.0–1.92	m + f	925	**0.21**	0.68	0.32	7.10 (7.04; 7.15)
		2.0–3.92	m + f	1461	**0.21**	1.00	0.61	9.76 (9.72; 9.80)
	NHANES [22]	1.0–3.0	m + f	798	**0.21**	0.53	0.67	31.65 (31.53; 31.76)

EAR, estimated average requirement; VELS, food composition survey to determine food intake by infants and small children for the estimation of acute toxicity risk from pesticide residues; FITS, feeding infants and toddlers study; NHANES, National Health and Nutrition Examination Survey; m, male; f, female.

**Table 4:** Mean vitamin E intake (mg/day) and prevalence of inadequate intake (% below EAR) in Brazil, Germany Russia and the United States.

Country	Study Name	Age Range (year)	Sex	*n*	EAR (mg/day)	Mean (mg/day)	SD (mg/day)	% below EAR (95% CI)
Brasil	Nutri Brazil Infantia [18]	1.0	m + f	58	5.0	4.16	1.55	no assessment possible
		2.0	m + f	657	5.0	4.76	2.77	no assessment possible
		3.0	m + f	974	5.0	4.89	4.31	no assessment possible
Germany	VELS [19,20]	1.0–1.9	m	81	**5.0**	4.65	3.37	54.14 (52.91; 55.36)
		1.0–1.9	f	87	**5.0**	4.90	3.51	51.14 (49.99; 52.28)
		2.0–2.9	m	89	**5.0**	5.91	3.87	40.70 (39.61; 41.80)
		2.0–2.9	f	85	**5.0**	5.54	5.09	45.78 (44.61; 46.94)
		3.0–3.9	m	71	**5.0**	6.67	4.65	35.97 (34.62; 37.32)
		3.0–3.9	f	74	**5.0**	5.90	3.98	41.05 (39.73; 42.38)
Russia	Healthy development from the first days of life [16]	1.0–2.0	m + f	1404	**5.0**	n.a.	n.a.	no assessment possible
USA	FITS [17]	1.0–1.92	m + f	925	**5.0**	5.00	3.04	50.00 (49.89; 50.11)
		2.0–3.92	m + f	1461	**5.0**	8.00	7.64	34.73 (34.66; 34.79)
	NHANES [22]	1.0–3.0	m + f	798	**5.0**	4.00	3.96	59.97 (59.85; 60.09)

EAR, estimated average requirement; VELS, food composition survey to determine food intake by infants and small children for the estimation of acute toxicity risk from pesticide residues; FITS, feeding infants and toddlers study; NHANES, National Health and Nutrition Examination survey; m, male; f, female, n.a., not available.

**Table 5:** Mean vitamin D intake (µg/day) and prevalence of inadequate intake (% below EAR) in Brazil, Germany, Russia and the United States.

Country	Study Name	Age Range (year)	Sex	*n*	EAR (µg/day)	Mean (µg/day)	SD (µg/day)	% below EAR (95% CI)
Brasil	Nutri Brazil Infantia [18]	1.0	m + f	58	**10.0**	4.52	2.56	no assessment possible
		2.0	m + f	657	**10.0**	5.24	4.26	no assessment possible
		3.0	m + f	974	**10.0**	5.51	5.87	no assessment possible
Germany	VELS [19,20]	1.0–1.9	m	81	**10.0**	n.a.	n.a.	no assessment possible
		1.0–1.9	f	87	**10.0**	n.a.	n.a.	no assessment possible
		2.0–2.9	m	89	**10.0**	n.a.	n.a.	no assessment possible
		2.0–2.9	f	85	**10.0**	n.a.	n.a.	no assessment possible
		3.0–3.9	m	71	**10.0**	n.a.	n.a.	no assessment possible
		3.0–3.9	f	74	**10.0**	n.a.	n.a.	no assessment possible
Russia	Healthy development from the first days of life [16]	1.0–2.0	m + f	1404	**10.0**	n.a.	n.a.	no assessment possible
USA	FITS [17]	1.0–1.92	m + f	925	**10.0**	8.0	3.04	74.47 (74.34; 74.56)
		2.0–3.92	m + f	1461	**10.0**	9.0	7.64	55.21 (55.14; 55.27)
	NHANES [21]	1.0–3.0	m + f	789	**10.0**	7.0	6.46	67.88 (67.77; 68.00)

EAR, estimated average requirement; VELS, food composition survey to determine food intake by infants and small children for the estimation of acute toxicity risk from pesticide residues; FITS, feeding infants and toddlers study; NHANES, National Health and Nutrition Examination Survey; m, male; f, female; n.a., not available.

**Table 6:** Mean folate intake (µg/day) and prevalence of inadequate intake (% below EAR) in Brazil, Germany, Russia and the United States.

Country	Study Name	Age Range (year)	Sex	*n*	EAR (µg/day)	Mean (µg/day)	SD (µg/day)	% below EAR (95% CI)
Brasil	Nutri Brazil Infantia [18]	1.0	m + f	58	**120.0**	414.54	157.96	3.11 (2.51; 3.71)
		2.0	m + f	657	**120.0**	444.80	189.84	4.35 (4.29; 4.42)
		3.0	m + f	974	**120.0**	466.20	270.88	10.06 (10.00; 10.12)
Germany	VELS [19,20]	1.0–1.9	m	81	**120.0**	97.33	64.69	63.70 (62.52; 64.88)
		1.0–1.9	f	87	**120.0**	97.56	62.67	63.99 (62.89; 65.08)
		2.0–2.9	m	89	**120.0**	122.66	88.06	48.80 (47.68; 49.91)
		2.0–2.9	f	85	**120.0**	116.61	128.74	51.05 (49.88; 52.22)
		3.0–3.9	m	71	**120.0**	121.00	77.34	49.48 (48.08; 50.89)
		3.0–3.9	f	74	**120.0**	120.89	190.06	49.81 (48.47; 51.16)
Russia	Healthy development from the first days of life [16]	1.0–2.0	m + f	1404	**120.0**	n.a.	n.a.	no assessment possible
USA	FITS [17]	1.0–1.92	m + f	925	**120.0**	352.00	145.99	5.60 (5.55; 5.65)
		2.0–3.92	m + f	1461	**120.0**	543.00	275.18	6.21 (6.18; 6.24)
	NHANES [22]	1.0–3.0	m + f	798	**120.0**	416.00	350.30	19.91 (19.81; 20.00)

EAR, estimated average requirement; VELS, food composition survey to determine food intake by infants and small children for the estimation of acute toxicity risk from pesticide residues; FITS, feeding infants and toddlers study; NHANES, National Health and Nutrition Examination Survey; m, male; f, female; n.a., not available.

**Table 7:** Mean calcium intake (mg/day) and prevalence of inadequate intake (% below EAR) in Brazil, Germany, Russia and the United States.

Country	Study Name	Age Range (year)	Sex	*n*	EAR (mg/day)	Mean (mg/day)	SD (mg/day)	% below EAR (95% CI)
Brasil	Nutri Brazil Infantia [18]	1.0	m + f	58	**500**	692.42	157.96	11.16 (10.07; 12.25)
		2.0	m + f	657	**500**	766.36	362.04	23.10 (22.97; 23.22)
		3.0	m + f	974	**500**	747.38	357.02	24.41 (24.33; 24.51)
Germany	VELS [19,20]	1.0–1.9	m	81	**500**	606.65	256.53	33.88 (32.72; 35.00)
		1.0–1.9	f	87	**500**	569.63	238.73	38.53 (37.42; 39.64)
		2.0–2.9	m	89	**500**	633.95	282.50	31.77 (30.73; 32.81)
		2.0–2.9	f	85	**500**	582.03	289.99	38.86 (37.72; 40.00)
		3.0–3.9	m	71	**500**	602.74	268.60	35.10 (33.76; 36.45)
		3.0–3.9	f	74	**500**	575.62	269.97	38.97 (37.66; 40.28)
Russia	Healthy development from the first days of life [16]	1.0–2.0	m + f	1404	**500**	699.20	374.40	29.73 (29.67; 29.80)
USA	FITS [17]	1.0–1.92	m + f	925	**500**	892.00	258.50	6.47 (6.42; 6.52)
		2.0–3.92	m + f	1461	**500**	910.00	340.16	11.40 (11.36; 11.44)
	NHANES [22]	1.0–3.0	m + f	798	**500**	972.00	1000.5	31.85 (31.74; 31.97)

EAR, estimated average requirement; VELS, food composition survey to determine food intake by infants and small children for the estimation of acute toxicity risk from pesticide residues; FITS, feeding infants and toddlers study; NHANES, National Health and Nutrition Examination Survey; m, male; f, female.

**Table 8:** Mean iron intake (mg/day) and prevalence of inadequate intake (% below EAR) in Brazil Germany, Russia and the United States.

Country	Study Name	Age Range (year)	Sex	*n*	EAR (mg/day)	Mean (mg/day)	SD (mg/day)	% below EAR (95% CI)
Brasil	Nutri Brazil Infantia [18]	1.0	m + f	58	**3.00**	10.18	3.54	2.13 (1.63; 2.62)
		2.0	m + f	657	**3.00**	11.02	4.59	4.03 (3.97; 4.09)
		3.0	m + f	974	**3.00**	11.36	4.40	2.87 (2.84; 2.91)
Germany	VELS [19,20]	1.0–1.9	m	81	**3.00**	5.87	2.77	15.01 (14.13; 15.89)
		1.0–1.9	f	87	**3.00**	5.77	3.09	18.50 (17.61; 19.39)
		2.0–2.9	m	89	**3.00**	6.65	3.15	12.33 (11.59; 13.06)
		2.0–2.9	f	85	**3.00**	6.01	2.63	12.62 (11.84; 13.40)
		3.0–3.9	m	71	**3.00**	6.86	2.70	7.64 (6.89; 8.39)
		3.0–3.9	f	74	**3.00**	6.20	2.83	12.91 (12.01; 13.81)
Russia	Healthy development from the first days of life [16]	1.0–2.0	m + f	1404	**3.00**	9.50	5.60	13.9 (11.84; 13.40)
USA	FITS [17]	1.0–1.92	m + f	925	**3.00**	10.30	3.95	3.23 (3.19; 3.27)
		2.0–3.92	m + f	1461	**3.00**	12.90	6.50	6.39 (6.35; 6.42)
	NHANES [22]	1.0–3.0	m + f	798	**3.00**	11.00	6.50	10.92 (10.84; 11.00)

EAR, estimated average requirement; VELS, food composition survey to determine food intake by infants and small children for the estimation of acute toxicity risk from pesticide residues; FITS, feeding infants and toddlers study; NHANES, National Health and Nutrition Examination Survey; m, male; f, female.

**Table 9:** Mean zinc intake (mg/day) and prevalence of inadequate intake (% below EAR) in Brazil Germany, Russia and the United States.

Country	Study Name	Age Range (year)	Sex	*n*	EAR (mg/day)	Mean (mg/day)	SD (mg/day)	% below EAR (95% CI)
Brasil	Nutri Brazil Infantia [18]	1.0	m + f	58	**2.50**	8.21	2.98	2.77 (2.20; 3.33)
		2.0	m + f	657	**2.50**	8.64	3.56	4.23 (4.17; 4.29)
		3.0	m + f	974	**2.50**	8.81	3.46	3.41 (3.37; 3.45)
Germany	VELS [19,20]	1.0–1.9	m	81	**2.50**	4.65	1.55	8.27 (7.59; 8.95)
		1.0–1.9	f	87	**2.50**	4.68	2.12	15.19 (14.37; 16.01)
		2.0–2.9	m	89	**2.50**	5.27	2.00	8.30 (7.69; 8.92)
		2.0–2.9	f	85	**2.50**	4.95	1.61	6.40 (5.83; 6.98)
		3.0–3.9	m	71	**2.50**	5.54	2.03	6.71 (6.01; 7.42)
		3.0–3.9	f	74	**2.50**	5.01	1.81	8.28 (7.53; 9.02)
Russia	Healthy development from the first days of life [16]	1.0–2.0	m + f	1404	**2.50**	n.a.	n.a.	no assessment possible
USA	FITS [17]	1.0–1.92	m + f	925	**2.50**	7.20	2.13	1.37 (1.34; 1.39)
		2.0–3.92	m + f	1461	**2.50**	9.70	4.59	5.84 (5.81; 5.87)
	NHANES [22]	1.0–3.0	m + f	798	**2.50**	8.30	6.22	17.55 (17.46; 17.65)

EAR, estimated average requirement; VELS, food composition survey to determine food intake by infants and small children for the estimation of acute toxicity risk from pesticide residues; FITS, feeding infants and toddlers study; NHANES, National Health and Nutrition Examination Survey; m, male; f, female; n.a., not available.

## 4. Discussion

### 4.1. Summary of Main Findings

Our cross-national comparisons suggest that inadequate intakes of key nutrients are prevalent across all four countries. In countries where data were available, a prevalence of inadequate intakes higher than 20% was found for vitamin A, D, E and calcium. In Germany folate intake was also found to be inadequate. Due to the limited availability of representative studies, our data only indicate that inadequate micronutrient intake in toddlers might be a global challenge affecting also affluent countries.

### 4.2. Interpretation and Comparison with Previous Research

In our review, inadequate vitamin A intakes were identified for Germany and Russia using the EAR cut-point method. However, we saw an inconsistent pattern in the United States: vitamin A intakes below the EAR were found in fewer than 10% of toddlers in the FITS study and in more than 30% of toddlers in the NHANES. One explanation for this discrepancy might be that the variability of vitamin A intake is high and an assessment of more than 40 days would be necessary to estimate accurately the average intake of an individual [23]. Conflicting results on vitamin A intake were also found in earlier studies. For example, the results of a nationwide Russian survey showed that mean vitamin A intake was below the recommended level not only in children (0 to 6 years) but also in all other age groups [24]. However, in European adolescents aged 10–19 years vitamin A intake was found to be in the acceptable range [25]. Moreover, the European Food Safety Authority (EFSA) concluded that vitamin A intake seemed to be adequate in European children [26]. Therefore, our findings concerning the intake adequacy on vitamin A should be considered in the light of these potential limitations. To draw clear conclusions on vitamin A intake in the future, it is necessary to develop more precise methods to assess vitamin A intake.

In addition, the results from our study suggest that vitamin E intake is inadequate in healthy toddlers in Germany and in the United States. Similar observations were made in European boys aged 10–14 years [25] and in a representative sample of adolescents aged 14–18 years from Sao Paulo [27]. These findings deserve further attention because vitamin E plays an essential role in the human body as a lipid-soluble, chain-breaking antioxidant [28,29]. Previous work, however, has called into question the utility of EAR values for vitamin E set by the IOM because reported intake appears to have a limited correlation with plasma levels [30]. Future research should focus on the accuracy of the EAR for vitamin E in healthy toddlers to allow clear conclusions on the adequacy of vitamin E intake to be drawn.

With regard to vitamin D, an evaluation of intake adequacy was only possible for the United States. For both studies, FITS and NHANES prevalence of inadequate intake was found to be high (more than 50% below EAR). This finding is consistent with previous work conducted in the United States, which showed that fewer than 2% of the population in all age groups met the Recommended Dietary Allowance (RDA) for this nutrient [31]. Because in the United States foods such as milk, juices and breakfast cereals are commonly fortified with vitamin D, similar or even higher prevalences of inadequate intake might be present for toddlers from the remaining countries, where no such fortification strategies exist. As data on vitamin D intake are missing for Brazil and Russia, studies should be conducted to get a valid picture on vitamin D intake in healthy toddlers from these countries. For Germany median intake values of vitamin D are available for healthy toddlers. In the German Representative Study of Toddler Alimentation (GRETA) median values ranged between 2.3 µg/day for 13–18 months old and 1.1 µg/day for 25–36 months old toddlers [32]. For the VELS study similar median values were reported for 1–3 year old (1.2 µg/day for males; 1.1 µg/day for females) [19]. It is not possible to evaluate the level of inadequacy for median values using the EAR cut-point method but the authors of the respective studies stated that vitamin D intake was low compared to recommendations [19,32]. In Germany supplementation of vitamin D is recommended for infants from the first week of life for the next 12 to 18 months depending on season of birth [33]. Data of the GRETA study show that 74% of the infants aged 10–11 months received vitamin D supplementation [34]. However, supplementation rates declined with age and even for the 12 months aged toddlers significant lower percentages were seen [34]. In addition, the EFSA concluded that dietary intakes of vitamin D are low in infants and young children living in Europe [26]. Similar results were reported for Brazil, where a population-based health survey showed an inadequate vitamin D intake for almost 100% of participants ≥12 years of age [35]. Although no data on vitamin D intake were available for toddlers from Russia, a Russian study of children aged three to six years reported vitamin D intake values (0.6 to 4.1 μg/day) below recommendations [36]. Because vitamin D is primarily produced by endogenous synthesis in the skin initiated by sunlight exposure [37], it may be inadequate during winter months, especially if dietary intake is low [26]. Taken together, these findings suggest that inadequate vitamin D intake is a problem which starts in early childhood and continues to be prevalent throughout life. Given the benefits of vitamin D for bone health and in view of results from earlier studies indicating that an inadequate vitamin D status might be associated with infections and autoimmune diseases [38,39,40,41,42], public health initiatives focusing on adequate vitamin D intake should be considered early in life.

Considering the dietary intake of folate, no consistent pattern could be identified across countries. While folate intake was found to be inadequate for up to 64% of German toddlers, this was the case in fewer than 10% of Brazilian and US toddlers (FITS only). One explanation for these cross-national differences might be the implementation of policies in Brazil and the United States which require fortification of grain products with folic acid [43,44]. For example, 100 g of wheat or maize flour must be fortified with 150 µg of folic acid in Brazil and 100 g of all cereal grains must be fortified with 140 µg of folic acid in the United States [43,44]. Such food fortification with folate is not very common across Europe [45], which might explain inadequate intakes found also in older age groups from this region. In Germany, for example, recommended intakes for folate were not reached in a representative sample of children and adolescents aged 6–17 years [46]. Moreover, according to the Healthy Lifestyle in Europe by Nutrition in Adolescence (HELENA) study, recommended intakes for folate were also not attained in older children in other European countries [25]. As folate plays an essential role in DNA synthesis and is important for optimal cell division and tissue growth [26], initiatives to improve folate intake starting early in childhood and continuing through adult age, especially in European countries, should be considered.

Adequate dietary calcium intake in childhood is important for normal skeletal growth, the mineralization of bones and teeth and the optimal achievement of peak bone mass, which is a precondition for the prevention of osteoporosis [47]. In our study, inadequate calcium intake could be demonstrated for Brazilian, German and Russian toddlers. While in the NHANES study inadequate intakes were also seen for toddlers from the United States, this was not the case for toddlers in the FITS study. Previous work reports inadequate calcium intakes for older age groups in all four countries. In the United States, only about 20% of females aged 9–18 and ≥51 years reached the recommendations from diet alone [48]. In a European sample of adolescents, inadequate calcium intake was found to be prevalent mainly in girls [25]. The prevalence of calcium inadequacy was >70% in both male and female participants older than 12 years in a population-based survey from Sao Paulo [35]. In Russia and Germany, intakes were also reported to be below recommendations in younger age groups. For Russian children aged 2–6 years, studies from Western Siberia and the Far North showed calcium intakes lower than recommended [36,49]. In a representative sample of German children aged 6–11 years, recommended calcium intake was also not observed [46]. Findings from previous work and the current study support the need for initiatives to ensure adequate calcium intake from early childhood onward to achieve optimal bone health.

With regard to iron and zinc mean intake values were found to be adequate across all four nations besides Russia, where no information on zinc intake was available for healthy toddlers. While the adequate intake values of zinc are consistent with the existing literature [8,26], opposite findings have been reported for iron within this age group. An actual systematic review on iron intake in children aged 6–36 months from Europe revealed a range of inadequate intakes of 10% to 50% below EAR [50]. However, this might be due to the application of different EAR values.

While we applied an EAR of 3.0 mg/day according to the IOM, Eussen *et al.* used an EAR of 5.3 mg/day set by the UK Department of Health [50,51]. They also included the data of the German VELS study and applying the UK EAR resulted in much higher inadequacies (range: 33.1% to 43.6% below EAR). However, the review also showed that iron deficiency anemia was below 5% in toddlers from Western Europe including Germany [50]. In addition, a German intervention study in breastfed infants revealed that a dietary iron intake about 25% below the German intake recommendation did not result in an increased risk for iron deficiency [32,52]. Considering these findings more research on the influences of dietary iron intake on iron metabolism in healthy toddlers is necessary [32] to improve the evidence base where iron intake recommendations can built on.

### 4.3. Strengths and Limitations

To our knowledge, this is the first review focusing on nutrient intake patterns in healthy toddlers from some of the world’s most populous nations, which are currently at differing stages of socioeconomic development. Given the importance of adequate early nutrition to future health, our review provides valuable information on available nutrient intake data and highlights existing research and data gaps for this age group. We applied several strategies to reduce the potential for publication bias, including a search and review of both published and unpublished “grey” literature and a search strategy which identified studies published in languages other than English. In addition, we tried to receive original data by contacting the authors directly.

Despite these important strengths, several potential limitations must be acknowledged. First, the comparability of the identified studies was limited by the heterogeneity of the reported variables; studies differed, for example, in investigated subgroups with regard to age, sex, time of data collection and nutrients reported. However, for most nutrients data could be compared among at least three out of the four countries. In addition, we considered the likelihood that seasonal effects might have influenced our results to be small as most of the data were collected throughout the year. Second, cross-country comparisons were complicated by different dietary assessment methods and -programs used to calculate the nutrient intake. Third, supplement intake was only assessed in the FITS study, potentially resulting in an overestimation of the prevalence of an inadequate intake in our study. However, supplement intake was reported to contribute less than 1% to the overall daily nutrient intake of the population of interest in the FITS study, and therefore the effect on the results is considered to be low [30]. Fourth, although our results are based on data from published articles and other publicly available sources, we were able to apply a modified version of the EAR cut-point method recommended by the EURRECA Network [14] by assuming that requirement distributions were symmetric and by excluding data on nutrient intake which were reported to be non-normally distributed. As we cannot completely rule out the possibility that other nutrient intake data were skewed and could have influenced prevalence estimates [14,53], we tried to overcome potential bias by using a more tolerant threshold as was done in previous research [14]. Fifth, it was beyond the scope of our review to give a global overview on the nutrient intake of healthy toddlers. Therefore, it is important that future reviews explore toddlers’ nutrient intake in other world regions like Africa and Asia to complete the worldwide picture. Sixths, although we aimed to identify studies which were nationally representative, recruitment strategies of the individual studies varied. For example, the Nutri Brasil Infância used a convenience sample of children attending preschools fulltime in nine Brazilian cities. Intake values observed in toddlers from rural areas [54] and in those not attending preschools [55] were notably lower than the values reported in Nutri Brasil Infância. As a result, the true prevalence of inadequate nutrient intakes at the population level is likely to be higher in Brazil. In addition, the nature of the data precluded us from examining potential associations between socioeconomic or educational factors and the toddlers’ nutrient intake. Therefore, studies focusing on such associations in the age group of healthy toddlers are urgently needed. Taken together the use of representative samples and standardized methods to assess the dietary intake are necessary to improve the comparability of cross-national data.

## 5. Conclusions

The results of our study indicates that potential inadequacies of key nutrients in healthy toddlers are prevalent across four populous nations independent of their current level of socioeconomic development. However, given the limited availability of comparable data for this age group across these nations, researchers worldwide need to work together both to combine and compare existing data and to identify and fill in data gaps. Results of such synergistic approaches will help to get a clear picture of the worldwide micronutrient status in healthy toddlers. This is necessary to support pediatricians, nutritionists and public health professionals in developing strategies to ensure optimal micronutrient intake from the first years of life.

## Supplementary Files

Supplementary File 1
