# Supplementary material for: Micronutrient Intake in Healthy Toddlers: A Multinational Perspective
Source: Nutrients. 2015 Aug 18;7(8):6938–55. doi: 10.3390/nu7085316 (PMC4555155; doi:10.3390/nu7085316)
Supplement: Supplementary File 1 [file nutrients-07-05316-s001.docx]

Supplementary Information

Detailed Information on the Search Strategies and Search Queries for Each Country

Brazil

In order to identify national studies on nutritional status of Brazilian toddlers, we first searched the Pubmed/Medline database for original articles. We used MeSH and free text terms including the keywords “child, preschool”, “nutrition assessment”, “nutritional status” and “Brazil”. The following search query was used: *("child, preschool"[MeSH Terms] OR ("child"[Text Word] AND "preschool"[Text Word]) OR "preschool child"[Text Word] OR "children"[Text Word] OR "toddlers"[Text Word]) AND ("nutrition assessment"[MeSH Terms] OR "nutritional status"[MeSH Terms]) AND ("Brazil"[MeSH Terms] OR "Brazil"[Text Word]) AND (“2000/01/01”[PDat] : “2014/01/15”[Pdat]).* The search on 15 January 2014 provided 301 hits.

We also searched the LILACS database containing articles published in Portuguese. We used the following search query: (mh:"child, preschool" OR mh:infant OR tw:"preschool child" OR tw:toddler OR (tw:child AND tw:preschool) OR mh:pre-escolar OR tw:pre-escolar OR mh:lactente OR tw:lactente) AND (mh:"nutritional status" OR mh:"nutrition assessment" OR mh:"alivacao nutricional" OR mh:"estado nutricional") AND (mh:Brazil OR tw:Brazil OR mh:Brasil OR tw:Brasil) AND year_cluster:("2000" OR "2001" OR "2002" OR "2003" OR "2004" OR "2005" OR "2006" OR "2007" OR "2008" OR "2009" OR "2010" OR "2011"). The search on 15 January provided 139 hits. After exclusion of duplicates that were referenced in both databases, 351 abstracts in English or Portuguese were screened for eligible studies.

The reference lists of the screened articles were searched for further publications on this topic. We also searched for publications on the homepages of institutions that may have had conducted nutritional surveys in the population of interest, e.g. the Brazilian Ministry of Health (Ministério da Saúde), the Brazilian Institute of Geography and Statistics (Instituto Brasileiro de Geografia e Estatística), the Ministry of Social Development and Fight against Hunger (Ministério do Desenvolvimento Social e Combate a Fome), the National Health Surveillance Agency (Agência Nacional de Vigilância Sanitária). We also conducted a Google search for keywords in English and Portuguese to obtain further information regarding identified studies “pré-escolar”, “estado nutricional”, “estudo”.

Germany

We searched the PubMed/Medline database on 15 January 2014 to identify eligible studies providing information on the nutrient intake status of healthy toddlers (aged 1–3 years) in Germany. We used MeSH and free text terms including the keywords “child, preschool”, “nutrition assessment”, “nutritional status” and “Germany”. The following search query produced 78 hits: *(("child, preschool"[MeSH Terms] OR ("child"[Text Word] AND "preschool"[Text Word]) OR "preschool child" [Text Word] OR "children"[Text Word] OR "toddlers"[Text Word]) AND ("nutrition assessment"[MeSH Terms] OR "nutritional status"[MeSH Terms]) AND ("Germany"[MeSH Terms] OR "Germany"[Text Word]) AND (“2000/01/01”[PDat] : “2014/01/15”[Pdat])).*

A Google search was also conducted by entering keywords “Kleinkind”, “Ernährung”, “Ernährungsstatus”, “Studie” and “Deutschland”. For studies that were considered as eligible, we searched for additional information by entering the exact study name into the Google search engine. Reference lists of identified articles were checked to detect other articles of interest. We also searched for publications on the homepages of the “Robert Koch Institute” (RKI; central federal institution responsible for disease control), the “Research Institute of Child Nutrition, Dortmund” (FKE) and the German Nutrition Society (DGE).

Russia

We searched the Pubmed/Medline database for original articles, in order to identify studies on nutritional status of preschool children conducted in Russia. We used MeSH terms and free text. Following keywords were chosen from the MeSH thesaurus: “child, preschool”, “nutrition assessment”, “nutrition survey”, “nutritional status”, “Russia”. The following search query was used:

("child, preschool"[MeSH Terms] OR ("child"[Text Word] AND "preschool"[Text Word]) OR "preschool child"[Text Word] OR "toddlers"[Text Word]) AND ("nutrition assessment"[MeSH Terms] OR "nutritional status"[MeSH Terms]) AND ("russia"[MeSH Terms] OR "russia"[Text Word] OR "Russian Federation"[Text Word]). The search on 15 January 2014 provided 34 hits.

We also searched the electronic scientific database elibrary.ru (http://elibrary.ru/defaultx.asp), where more than 7,800 Russian scientific journals are referenced, and electronic catalogue “Medicine” (Accessed via Central Scientific Medical Library: http://193.232.7.102/cgiopac/opacg/opac.exe). We used following keywords in Russian: “дети раннего возраста”, “питание”, “пищевой статус” (“toddlers”, “nutrition”, “nutritional status”). The keywords were searched in all fields. The search on 15 January 2014 provided 7 hits. An additional search was conducted in Google.scholar by entering the keywords “дети раннего возраста”, “питание”, “пищевой статус”, “научное исследование”, “Россия” (“toddlers”, “nutrition”, “nutritional status”, “survey”, “Russia”). The reference lists of publications that appeared to be eligible were checked.

For all eligible studies, we entered the exact study names in the Google search engine, in order to obtain further information on the studies. Homepages of research institutes and organizations that conducted these studies or provided funding were also searched. We screened the homepages of the WHO (Global Database on Child Growth and Malnutrition), and of the Ministry of health protection of Russian Federation (Mинистерство здравоохранения Российской Федерации) to identify further research related to toddler’s nutrition.

United States

To identify studies on nutritional status of toddlers conducted in the USA, we first searched the PubMed/Medline database for original articles. Following keywords were chosen from the Medical Subject Heading (MeSH) thesaurus: “child, preschool”, “nutrition assessment”, “nutritional status”, “United States”. We searched these keywords as well as a free text term “toddler” in text fields (title and abstract). We restricted our search only on articles published from 1 January 2000 on. The following search query was used: *(“child, preschool”[MeSH Terms] OR (“child”[Text Word] AND “preschool”[Text Word]) OR “preschool child”[Text Word] OR “children”[Text Word] OR “toddlers”[Text Word]) AND (“nutrition assessment”[MeSH Terms] OR “nutritional status”[MeSH Terms]) AND (“United States”[MeSH Terms] OR “United States”[Text Word]) AND (“2000/01/01”[PDat] : “2014/01/15”[Pdat]).* The search on 15 January 2014 provided 1014 hits that were screened for eligible surveys. In addition, references of the articles providing indications on nationwide surveys were checked for further publications of interest. We also searched the websites of health or nutrition related societies in the USA, e.g., “the US Department of Agriculture and Health and Human Services”, “the National Institutes of Health”, “Centers for Disease Control and Prevention” and “the Academy of Nutrition and Dietetics” for eligible studies.
